# Analysis of Short-Term Effects of Air Pollution on Cardiovascular Disease Using Bayesian Spatio-Temporal Models

**DOI:** 10.3390/ijerph17030879

**Published:** 2020-01-31

**Authors:** Yi Liu, Jingjie Sun, Yannong Gou, Xiubin Sun, Dandan Zhang, Fuzhong Xue

**Affiliations:** 1 Department of Epidemiology and Biostatistics, School of Public Health, Shandong University, 44, Wenhuaxi Street, Jinan 250012, China; 2 Health and Family Planning Information Center of Shandong Province, 75, Yuhan Street, Jinan 250014, China

**Keywords:** Bayesian statistics, spatio-temporal models, air pollution, cardiovascular disease, China

## Abstract

There has been an increasing number of clinical and epidemiologic research projects providing supporting evidence that short-term exposure to ambient air pollution contributes to the exacerbation of cardiovascular disease. However, few studies consider measurement error and spatial effects in the estimate of underlying air pollution levels, and less is known about the influence of baseline air pollution levels on cardiovascular disease. We used hospital admissions data for cardiovascular diseases (CVD) collected from an inland, heavily polluted city and a coastal city in Shandong Province, China. Bayesian spatio-temporal models were applied to obtain the underlying pollution level in each city, then generalized additive models were adopted to assess the health effects. The total cardiovascular disease hospitalizations were significantly increased in the inland city by 0.401% (0.029, 0.775), 0.316% (0.086, 0.547), 0.903% (0.252, 1.559), and 2.647% (1.607, 3.697) per 10 μg/m^3^ increase in PM_2.5_, PM_10_, SO_2_, and NO_2,_ respectively. The total cardiovascular diseases hospitalizations were increased by 6.568% (3.636, 9.584) per 10μg/m^3^ increase in the level of NO_2_. Although the air pollution overall had a more significant adverse impact on cardiovascular disease hospital admissions in the heavily polluted inland city, the short-term increases in air pollution levels in the less polluted coastal areas led to excessive exacerbations of cardiovascular disease.

## 1. Introduction

Cardiovascular diseases (CVD) cause 17.7 million deaths every year (31% of all global deaths), and are the most common cause of death and disability around the world [1,2,3,4,5]. Many studies across the world have shown that ambient air pollution contributes to the increase of mortality, morbidity, and hospitalizations of CVD [6,7,8,9,10,11,12,13]. Without a precise control of the sources of pollution and the application of strict policies, air pollution has become one of the major public health concerns in China [14,15,16,17,18,19].

Researchers have previously conducted a series of analyses on the effect of ambient air pollution on CVD in China [20,21,22,23,24,25,26,27]; however, few studies have considered measurement error and spatial effects in the estimate of the underlying air pollution levels. Health data are generally only available in an aggregated form for an entire administrative area, but daily measurements of air pollution consist of concentrations from a set of different monitoring sites, meaning that a corresponding single daily measure of pollution is required as a predictor in the regression model. The most commonly used approach is to simply average the measurements over spatial locations, and to use the resulting summary in a health model. However, this simple summary ignores any measurement error or spatial variability in the underlying pollution field and, as such, subsequent estimates of the health effects may be biased [28]. Different baselines of air pollution concentration may lead to different health outcomes when there is a short-term increase in the air pollution level, a topic few existing studies have focused on.

The inland city of Jinan is located in a hilly area of Shandong province, East China. It is an industrial city that suffers from serious air pollution, largely due to the oil and coal burning from industrial processes and vehicle emissions. In contrast, the coastal city of Weihai is one of the least polluted regions in China. It has been named the “National Hygienic City” and the “National Garden City”. In 2003, the Weihai Municipal Government was awarded the “UN-Habitat Scroll of Honor”, for outstanding improvements in shelter and the urban environment [29].

In this study, we proposed a Bayesian spatio-temporal model to accurately estimate the underlying air pollution level in the study regions, then assessed the short-term effect of air pollutants, including PM_2.5_, PM_10_, SO_2_, and NO_2_, on the exacerbation of CVD by analyzing the data of hospital admissions collected from all the tertiary-level and secondary-level hospitals in Jinan and Weihai, both in Shandong Province. Based on the analysis results, we further looked into the baseline influence on the health effect of the air pollution. This study thus provides public health evidence for developing policies and health management strategies for different ambient pollution environments.

## 2. Materials and methods

### 2.1. Hospital Admissions

Hospital admission records for CVD between 1st January, 2014 and 31st December, 2016 were obtained from all tertiary-level and secondary-level hospitals in Jinan and Weihai. Each record contained the hospital name, date of service, age, gender, date of birth, current address, and disease ICD code (International Classification of Diseases, Tenth Revision [ICD-10] codes). Cardiovascular disease outcomes were considered, based on the ICD-10 codes: I00–99.

### 2.2. Air Pollution and Meteorological Data

**Figure 1:**
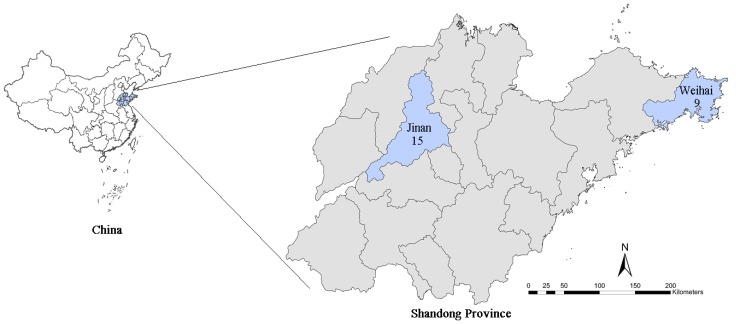
The location of Jinan and Weihai in the Shandong Province; the two cities are shown in blue. The numbers represent the number of air quality monitoring stations in each city.

The monitoring stations established by both the China National Environmental Protection Administration and Shandong local government were considered in this study. There were two sites excluded from the study in the city of Jinan, both of which were new monitoring stations established in May, 2015. Hence, in total, 15 and 9 stations in Jinan and Weihai, respectively, were included in the analysis, as shown in Figure 1. The maximum distance between monitors was 74.64 km and 87.64 km in Jinan and Weihai, respectively. Each air quality monitoring station provided hourly readings of PM_2.5_, PM_10_, SO_2,_ and NO_2_ levels, between 1st January, 2014 and 31st December, 2016. The meteorological data were obtained from the China Meteorological Administration Data Center (URL: https://www.data.cma.cn/). These data were collected daily and included the daily mean, and the highest and lowest values of the monitored objects. We considered the daily mean temperature, relative humidity, wind speed, and pressure as confounding factors of weather in the analysis.

### 2.3. Method of Analysis

In this paper, we proposed a two-stage approach: firstly, we modeled the underlying pollution concentrations using a Bayesian spatio-temporal model; secondly, we assessed their relationship with the health data, using a frequentist generalized additive model. We implemented the first stage Bayesian spatio-temporal model using a Markov chain Monte Carlo (MCMC) simulation. There were three parts of this hierarchical model:

(1) $$\ln(W_{st})∼N(Z_{t}+m_{s},\sigma_{\varepsilon }^{2})$$

(1) The measurement error level: We modeled the air pollution data on the log scale because the pollution concentrations were nonnegative and often skewed to the right. The combination of true underlying process $Z_{t}$ and spatial effect $m_{s}$ is linked with the observed pollution concentrations using a measurement error model, where the measurement errors are independent with zero mean and constant variance of $\sigma_{\varepsilon }^{2}$
  (2) $$\begin{array}{l} Z_{t}∼N(\mu +\rho (Z_{t-1}-\mu ),\sigma_{z}^{2})\\ m_{s}∼MVN(0,\sigma_{m}^{2}\Sigma (\phi )) \end{array}$$
(2) The underlying process level: The true levels were modeled by a first order autoregressive process which had variance $\sigma_{z}^{2}$ and lag one correlation coefficient ρ. The spatial structure was represented by a set of zero-mean Gaussian random effects $m_{s}$, which had variance $\sigma_{m}^{2}$ and correlation matrix Σ(ϕ). The latter was constructed using the Matern class of functions, with a smoothness parameter fixed at 0.5. This gave an exponential correlation structure $Corr[s,s^{\prime }]=\exp\left(-\left\|s-s^{\prime }\right\|\phi \right)$, where ‖.‖ denotes Euclidean distance.
  (3) $$\begin{array}{l} \sigma_{\varepsilon }^{2}∼Inverse-Gamma(0.01,0.01)\\ \sigma_{z}^{2}∼Inverse-Gamma(0.01,0.01)\\ \sigma_{m}^{2}∼Inverse-Gamma(0.01,0.01)\\ \phi ∼Discrete-Uniform\left(a_{\phi },b_{\phi }\right) \end{array}$$
(3) The parameter level: The spatial range parameter ϕ was assigned a discrete uniform prior with possible values ranging between the minimum and maximum distance between monitoring locations [30]. The variance parameters $\sigma_{\varepsilon }^{2}$, $\sigma_{z}^{2}$, and $\sigma_{m}^{2}$ were assigned weekly informative priors [31].

In order to draw inference using this Bayesian model, the MCMC algorithm was run for 40,000 iterations, discarding the first 10,000 as “burn in” samples. Finally, the posterior mean values were summarized as estimates of the true underlying air pollution levels and plugged into the second stage health models.

In the second stage, we applied generalized additive Poisson models in frequentist settings to examine the associations between air pollution (PM_2.5_, PM_10_, SO_2_, and NO_2_) for 0–6 day lags and day-to-day variations in the CVD hospital admissions for the total number of patients, stratified by sex and age. We also fitted multipollutant models to assess the stability of air pollution health effects, where daily air pollution exposures were included jointly, at the same lag as in the models. A quasi-Poisson distribution was adopted, in order to overcome the overdispersion of hospital admissions data. The smoothed functions captured the nonlinear relationship between daily hospital admissions and the time-varying covariates [32,33], while the degrees of freedom of natural splines were determined by the Akaike’s information criterion [34]; therefore, calendar time (7 df per year) and the meteorological variables, including temperature (4 df), relative humidity (4 df), wind speed (3 df), and pressure (5 df), were adopted. In addition, the day of the week (DOW) was also included as a dummy variable in the models. The MGVC package (v.1.8–17) [33] in R v.3.4.3 [35] was applied, to construct the health models. Then, all the results were presented as the percentage change in the relative risk (RR) of hospitalizations and its 95% confidence interval (CI) in association with a 10 μg/m^3^ increase in daily air pollutants.

## 3. Results

**Figure 2:**
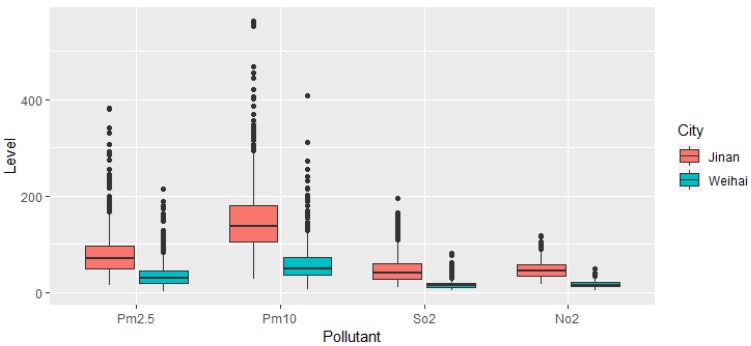
A boxplot of daily mean air pollution levels in Jinan and Weihai.

**Figure 3:**
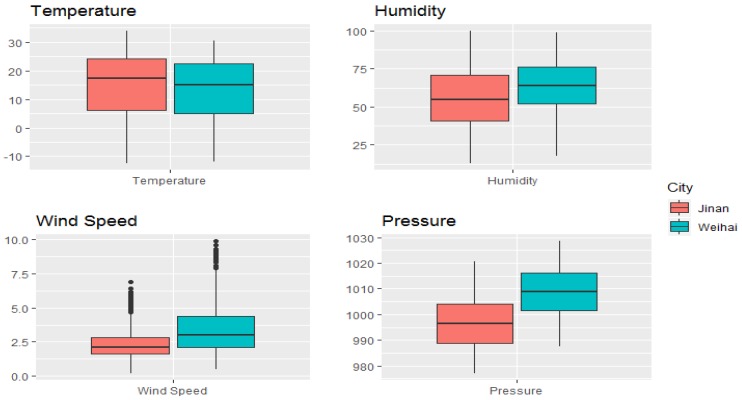
A boxplot of meteorological factors in Jinan and Weihai.

In this study, the number of CVD hospital admissions during the study period was 306,963 and 91,232 in Jinan and Weihai, respectively. In total, there were 219,121 male patients and 179,074 female patients. Moreover, there were 209,554 patients that were over 65 years old, and 188,641 records were for patients aged under 65. The details of the hospital admissions in the two cities can be found in Table 1. Figure 2 and Figure 3 present the boxplot of the daily mean air pollution levels and the meteorological factors during the study period in Jinan and Weihai. The daily maximum values of the air pollution concentrations in Jinan were above China’s ambient air quality secondary standards (PM_2.5_: 75 μg/m^3^, PM_10_: 150 μg/m^3^, SO_2_: 150 μg/m^3^, and NO_2_: 80 μg/m^3^) [36], while the daily maximum concentrations of SO_2_ and NO_2_ in Weihai were below the standard. There was an obvious spatial heterogeneity among the distribution of ambient air pollution levels, such that the pollutant measurements in the coastal city of Weihai were much lower than the ones in the inland city of Jinan.

**Figure 4:**
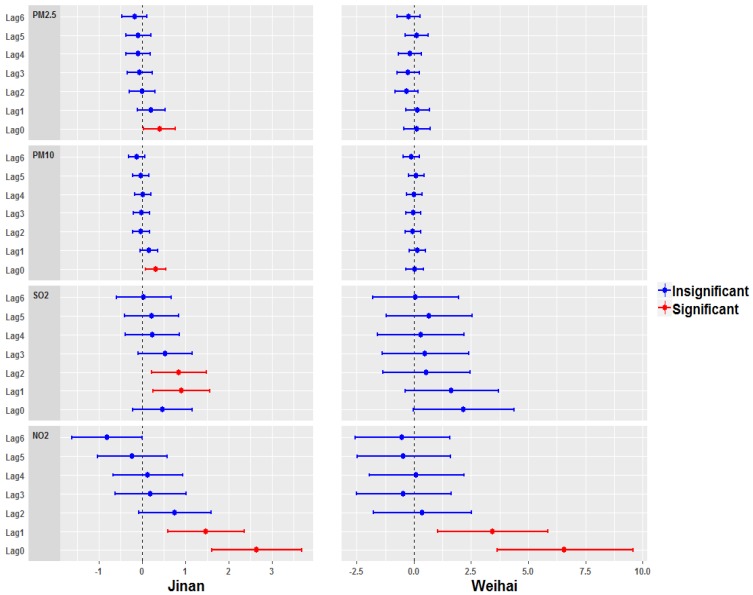
A plot of the percentage change in the relative risk of the total cardiovascular diseases hospitalizations with a 95% confidence interval (CI) per 10 μg/m^3^ increase in the air pollution levels, in the cities of Jinan and Weihai.

Figure 4 presents the results for lags over 0–6 days from the single-pollutant models, including the effect estimates and corresponding 95% confidence intervals (CIs) of the percentage changes of relative risk (RR) for the total CVD admissions per 10μg/m^3^ increase in PM_2.5_, PM_10_, SO_2_, and NO_2_ concentrations, in Jinan and Weihai. There were significant associations between all types of pollutants and the CVD admissions in Jinan. The total CVD hospitalizations significantly increased by 0.401% (0.029, 0.775), 0.316% (0.086, 0.547), 0.903% (0.252, 1.559), and 2.647% (1.607, 3.697) per 10μg/m^3^ increase in PM_2.5_, PM_10_, SO_2,_ and NO_2_, respectively. The health effects of air pollution were less dramatic in the coastal city of Weihai, although the total CVD hospitalizations increased by 6.568% (3.636, 9.584) per 10 μg/m^3^ increase in NO_2_ at lag 0.

The results of the multipollutant models are shown in Table 2. For total CVD hospitalizations in Jinan, the effect of PM_2.5_, PM_10,_ and SO_2_ become insignificant when adjusted for NO_2_. Then, the effect of PM_2.5_ and PM_10_ becomes insignificant when adjusted for each other. Moreover, the association between an increase in the levels of SO_2_ and NO_2_ and the total hospital admissions was consistent when adjusted for PM_2.5_ and PM_10_. For total CVD hospital admissions in Weihai, the effect of NO_2_ was consistent when adjusted for other pollutants, such that the total hospital admissions were significantly increased by 9.606% (5.887, 13.456), 8.453% (5.048, 11.97), and 8.415% (4.448, 12.532), per 10 μg/m^3^ increase in NO_2,_ when adjusted for PM_2.5_, PM_10,_ and SO_2_, respectively.

Table 3 shows the results of the subgroup analysis. Regarding gender, stronger effects were observed for females in Jinan, except for PM_2.5_, such that the significant effects of PM_2.5_ were 0.404% (0.009, 0.801) and 0.398% (0.013, 0.783) on male and female patients, respectively, both at lag 0. However, we found the opposite results in Weihai, where the air pollution had a stronger effect on male patients. For example, the SO_2_ effect on male patients was significant (2.859% (0.340, 5.441) at lag 0), while the effect on female patients was insignificant. In addition, the significant NO_2_ effects were 7.419% (4.031, 10.918) and 5.535% (2.070, 9.119) for male and female patients, respectively, at lag 0. Regarding age, the results varied between the two cities. The air pollution effects on the younger patients were stronger in Jinan, for example, the PM_2.5_ effect on aged patients in Jinan was insignificant, but the effect on patients under 65 years of age was significant: 0.485% (0.074, 0.899) at lag 0. In Weihai, the air pollution effects on the aged patients were more severe. The younger patients’ hospitalizations were significantly affected only by the increase in NO_2_ levels, but PM_2.5_, SO_2_, and NO_2_ effects on aged patients were significant. The NO_2_ effects were 5.236% (1.591, 9.011) and 7.612% (4.321, 11.006) on younger and aged patients, respectively.

## 4. Discussion

In this study, we assessed the effects of short-term exposure to ambient air pollution on the exacerbation of CVD, using hospital admissions data from a coastal and an inland city in Shandong province, China. Since the health data were collected from tertiary-level and secondary-level hospitals, all the patients went through a reasonable diagnosis and treatment during the admission time; therefore, the accuracy of diagnosis and the reliability of the datasets can be guaranteed.

The public health burden of the present exposure level is substantial. As air pollution is experienced by everybody, even a small increase in risk can be associated with very large numbers of people being ill [37,38,39]. It is therefore extremely important that the risks to health are estimated accurately, and for that to happen there needs to be accurate recording of the levels of exposure that might be experienced by populations at risk. In this analysis, a Bayesian spatio-temporal model was applied to obtain the underlying pollution level. This method effectively captures the spatial and temporal features of the ambient air pollution data, and also considers the measurement error term in the data. As a result, the corresponding estimated percentage change from the health model is more adequate than the ones obtained from the model when simply taking the average over all of the air pollution data collected at the monitoring stations in the same area.

There are likely to be computational considerations associated with jointly fitting the health and exposure models in Bayesian settings, especially if the latter uses large amounts of data over space and time. When the exposure model is complicated, or when one is interested in running multiple candidate epidemiological models with different sets of covariates, a single model is not going to provide an efficient method of investigation. We therefore adopted two-stage model settings in order to ease the computational burden of running a combined model (this has been adopted in a number of cases [28,40,41,42,43]). The two-stage approach has the advantage that the exposure model, which is likely to be the most computationally demanding, does not require a refit when running multiple health effect analyses. Moreover, we implemented the health models in a frequentist setting, as generalized additive models are the most commonly used models in epidemiological studies. The frequentist modeling obtained the confidence intervals of the estimates, instead of credible intervals deducted from a Bayesian method, which benefited us when comparing our results with the ones from other epidemiological studies.

We found that the associations between air pollution and CVD hospitalizations were spatially heterogeneous, and that air pollution contributed, overall, more prominent adverse health effects in the inland city. Jinan contains significant oil refineries and metallurgical and mechanical sectors and, moreover, vehicle emissions are one of the major causes of the heavy air pollution in the city. Furthermore, geographical factors are also influential, as Jinan is situated in a typical valley basin and is surrounded by the largest mountain group in East China, making it difficult for the emitted ambient pollution to drift away from the city and consequently leading to frequent cases of heavy smog.

In some cases, we found that the adverse health effect is smaller in a highly polluted area when the air pollution level goes up dramatically in a short period of time—this is consistent with the results from previous studies [24,27,44,45]. We found that only the effect of an increase of 10 μg/m^3^ of NO_2_ (lag 0) on CVD hospital admissions was significant in the coastal city of Weihai. Comparing the effects of NO_2_ in the two cities, we noticed that the effect in Weihai was much stronger than that in Jinan, being 6.568% (3.636, 9.584) in Weihai and 2.647% (1.607, 3.697) in Jinan. However, Figure 2 shows that the median NO_2_ concentration in Weihai during the study period was 16, and for Jinan was 44. This might be because in cities with higher air pollution such as Jinan, the public are less sensitive to air pollution, as their body functions, especially the underlying biochemical processes, may have become adjusted to exposure to a high level of the toxic component. Moreover, the estimates for NO_2_ remained significant after adjustment for other pollutants, suggesting that NO_2_ is important for the air pollution mixture in these two cities.

The subgroup analysis also shows the spatial heterogeneity. For example, the adverse effects of air pollution on the hospitalizations of younger patients were stronger than the aged patients in Jinan, but the results were opposite in Weihai. This could be caused by aged people actively adopting defensive measures when living in heavily polluted cities, such as purchasing air filters, reducing outdoor activities, or wearing masks. On the contrary, young people have more outdoor activities and are more likely to be exposed to air pollution, especially if they are in the construction or manufactural industry. In contrast, people that live in less polluted regions, such as Weihai, may have a very weak sense of air pollution self-protection and precautions, which could expalin why the air pollution effect on aged patients was stronger.

This study has a limitation. We excluded two monitoring sites from the study in the city of Jinan, which were both newly established in May, 2015. Since our study period was from 2014 to 2016, the missing rates of these two sites were about 44%. Given that these missing values appeared sequentially since the beginning of the study period, the accuracy of the predicted values using the Bayesian method may be an issue. In our study, we firstly integrated the spatial and temporal information to obtain the underlying air pollution levels, then plugged such values into the health models to estimate the health effects. Undoubtedly, the error introduced through predictions in the first stage can lead to bias in the estimate of the health effects; therefore, we need to apply a more efficient modeling method to predict this type of missing data in future studies.

## 5. Conclusions

Our analysis showed that air pollution was significantly associated with CVD hospital admissions in the inland city, but the short-term increases in air pollution levels, especially NO_2_, in the less polluted coastal city can also lead to the excessive exacerbation of CVD. Therefore, we suggest that not only the public living in heavily polluted cities need defensive activities but that the people inhabiting less polluted cities should also adopt self-protection measures when there is an obvious incline in the air pollution in a short time period.

## Figures and Tables

**Table 1:** The daily hospital admissions grouped by sex, age, and sub-disease in the cities of Jinan and Weihai (from 1st January, 2014 to 31st December, 2016).

City	Hospitalization (Cases/Per Day)	Min	P25	P50	P75	Max
	Total	8	195	291	346	580
	Male	5	106	158	191	308
Jinan	Female	3	89	131	157	274
	≥65	3	103	148	180	361
	<65	5	92	138	169	296
	Total	1	67	80	96	200
	Male	0	37	45	54	113
Weihai	Female	1	29	36	43	89
	≥65	0	37	45	54	109
	<65	1	28	35	44	104

**Table 2:** The percentage change in the relative risk of the total cardiovascular disease hospitalizations with a 95% confidence interval (CI) per 10 μg/m^3^ increase in the air pollution levels estimated from multipollutant models, where * indicates statistically significant estimates (*p* < 0.05).

**Jinan**							
**PM_2.5_**		**PM_10_**		**SO_2_**		**NO_2_**	
Single pollutant	0.401 (0.029, 0.775) *	Single pollutant	0.316 (0.086, 0.547) *	Single pollutant	0.903 (0.252, 1.559) *	Single pollutant	2.647 (1.607, 3.697) *
+PM_10_	−0.279 (−1.106, 0.555)	+PM_2.5_	0.472 (−0.045, 0.992)	+PM_2.5_	0.889 (0.156, 1.628) *	+ PM_2.5_	3.164 (1.835, 4.509) *
+SO_2_	0.360 (−0.057, 0.778)	+SO_2_	0.307 (0.050, 0.565) *	+PM_10_	0.859 (0.115, 1.609) *	+ PM_10_	3.018 (1.631, 4.423) *
+NO_2_	−0.295 (−0.764, 0.177)	+NO_2_	−0.124 (−0.429, 0.183)	+NO_2_	0.298 (−0.584, 1.188)	+ SO_2_	3.879 (2.483, 5.295) *
+All	−0.342 (−1.181, 0.504)	+All	0.071 (−0.475, 0.620)	+All	0.316 (−0.568, 1.208)	+All	4.229 (2.564, 5.922) *
**Weihai**							
**PM_2.5_**		**PM_10_**		**SO_2_**		**NO_2_**	
Single pollutant	0.161 (−0.356, 0.680)	Single pollutant	0.143 (−0.210, 0.497)	Single pollutant	2.149 (−0.031, 4.377)	Single pollutant	6.568 (3.636, 9.584) *
+PM_10_	−0.001 (−0.808, 0.812)	+PM_2.5_	0.144 (−0.409, 0.699)	+PM_2.5_	3.580 (0.472, 6.785) *	+PM_2.5_	9.606 (5.887, 13.456) *
+SO_2_	−0.239 (−0.949, 0.477)	+SO_2_	−0.021 (−0.447, 0.407)	+ PM_10_	2.875 (0.264, 5.554) *	+PM_10_	8.453 (5.048, 11.970) *
+NO_2_	−0.536 (−1.204, 0.136)	+NO_2_	−0.167 (−0.582, 0.249)	+NO_2_	−1.980 (−4.767, 0.887) *	+SO_2_	8.415 (4.448, 12.532) *
+All	−0.654 (−1.597, 0.298)	+All	0.098 (−0.457, 0.656)	+All	0.035 (−3.277, 3.460)	+All	9.648 (5.518, 13.940) *

**Table 3:** The changes in the relative risk of cardiovascular disease hospitalizations for patients by gender and age, and their 95% confidence interval (CI) per 10 μg/m^3^ increase in air pollutants in Jinan and Weihai, where * indicates statistically significant estimates (*p* < 0.05).

		Jinan		Weihai	
Pollutant	Class	Estimate	Lag	Estimate	Lag
PM_2.5_	Male	0.404 (0.009, 0.801) *	0	−0.419 (−0.992, 0.157)	2
	Female	0.398 (0.013, 0.783) *	0	−0.420 (−1.010, 0.174)	3
	≥65	0.323 (−0.056, 0.704)	0	0.660 (0.109, 1.214) *	5
	<65	0.485 (0.074, 0.899) *	0	−0.577 (−1.198, 0.048)	3
PM_10_	Male	0.308 (0.063, 0.553) *	0	0.119 (−0.260, 0.499)	3
	Female	0.326 (0.088, 0.565) *	0	0.283 (−0.138, 0.706)	1
	≥65	0.279 (0.045, 0.514) *	0	0.306 (−0.068, 0.682)	5
	<65	0.356 (0.100, 0.612) *	0	−0.257 (−0.684, 0.172)	4
SO_2_	Male	0.749 (0.058, 1.445) *	1	2.859 (0.340, 5.441) *	0
	Female	1.089 (0.415, 1.768) *	1	1.281 (−1.292, 3.921)	0
	≥65	0.709 (0.041, 1.381) *	1	2.438 (0.344, 4.576) *	5
	<65	1.116 (0.398, 1.839) *	1	1.924 (−0.807, 4.730)	0
NO_2_	Male	2.516 (1.414, 3.630) *	0	7.419 (4.031, 10.918) *	0
	Female	2.803 (1.725, 3.892) *	0	5.535 (2.070, 9.119) *	0
	≥65	2.284 (1.225, 3.355) *	0	7.612 (4.321, 11.006) *	0
	<65	3.033 (1.880, 4.199) *	0	5.236 (1.591, 9.011) *	0
